# Sexuality in Ageing Male: Review of Pathophysiology and Treatment Strategies for Various Male Sexual Dysfunctions

**DOI:** 10.3390/medsci7100098

**Published:** 2019-09-20

**Authors:** Eric Chung

**Affiliations:** 1 AndroUrology Centre, Brisbane, QLD 4000, Australia; ericchg@hotmail.com; Tel.: +617-38321168; 2 University of Queensland, Princess Alexandra Hospital, Brisbane, QLD 4000, Australia; 3 Macquarie University Hospital, Sydney, NSW 2109, Australia

**Keywords:** sexuality, ageing, sexual function, erectile function, ejaculation, orgasm, sexual desire

## Abstract

Sexual function among older men is often thought to decrease as part of normal ageing biology despite the fact that sexuality remains an important issue in the elderly. Sexual dysfunction in the aged male is likely multifactorial in nature, with the development and/or progression of medical comorbidities often resulting in decline in male sexual function and poor treatment response. At present, sexual dysfunction in the ageing male is poorly investigated and understood, and current treatment strategies aim at improving sexual desire and erectile function with limited data on ejaculatory and orgasmic dysfunctions. In addition, men are often reticent to seek help for health concerns including sexual dysfunction. The following article provides a narrative review of strategies to address various aspects of sexual dysfunction in the ageing male. Clinicians need to be educated to be sensitive when discussing sexuality issues among older men and to present practical solutions that take into account individual and cultural differences.

## 1. Introduction

The population is ageing and the rate at which the population ages is likely to increase over the next three decades. It is estimated that the number of older persons aged 60 years and over, will double in population to more than two billion by year 2050 in the world [1]. This will have major social and economic consequences with the ageing population, placing considerable burden to the current public healthcare system.

While a loss of sexuality could be considered normal and inevitable with ageing process, sexuality remains a key facet of masculinity, and in part of how men define themselves that sits apart from chronological age. Despite the perception of an asexual older age, older men continue to enjoy a robust sex life. This general misperception and prejudices of an asexual old age may be related to myths regarding older people and their sexual activities, difficulties experienced by older patients in disclosing sexual problems to healthcare professionals, and that sexual health may be less important in the overall scheme of their medical comorbidities. Men in particular are often hesitant to seek help for health concerns including sexual dysfunction. Furthermore, there is a lack of understanding among healthcare professionals about sexual health in older patients, compounded by short medical consultation time and limited knowledge or training to deal with sexual issues [2].

With longer life expectancy, more men are seeking to preserve their sexuality into their old age. Age is no longer thought to be a deterrent to an active sex life, but the health problems experienced by individuals (or their partners) may lead to reprioritising the value placed on sex [2,3]. The concept “sexually active life expectance” denotes the amount of remaining years for a person to remain sexually active [3]. In general, while men have a longer sexually active life expectance than for women, many men lose more years of sexually active life as a result of poorer health.

It is thought that a loss of sexuality in older men was natural and inevitable with ageing, and little attention was given to sexuality, sexual function and treatment of various male sexual dysfunctions in the older population. Male sexual function can be classified into four broad domains, namely, sexual desire, penile erection, ejaculatory and orgasm functions. This mini review will focus on various male sexual dysfunctions and provide a review on various pathophysiology mechanisms as well as treatment strategies to deal with sexual dysfunctions in ageing men (see Appendix A).

## 2. Erectile Dysfunction

The presence of erectile dysfunction (ED), defined as the persistent inability to attain and maintain penile erection sufficient for sexual intercourse [4], is the commonest reason for older men being sexually inactive. A normal penile erection is pivotal for men to be sexually active. Men with ED often experience considerable psychosexual stressors and relationship problems as well as reporting a loss of their self-confidence, further exacerbating underlying ED.

Various large-scale studies confirmed the close relationship between ED and ageing men. The Massachusetts Male Aging Study (MMAS) reported that ED was present in more than 50% of participants and that the prevalence of severe dysfunction tripled from 5% to 15% between the subjects aged 40 and 70 years [5]. Similar findings were echoed by the European Male Aging Study (EMAS) with more than 30% of the entire EMAS sample reporting ED and that the prevalence of the condition was reported to be higher in the older age groups, peaking in men 70 years and older (64%) [6]. With the rapidly expanding ageing population and higher life expectancy, it is estimated that the prevalence of ED will reach more than 300 million worldwide by 2025 [7]. At the same time, the severity of ED also increases with advancing age, a reflection of the increasing medical morbidities in ageing men such as the presence of hypertension, vascular disease, diabetes, hypogonadism, chronic renal disease, depression and many others, which contribute to adverse changes in neurohumoral pathways, a decreased cavernosal blood flow and subsequent penile fibrosis [8]. The use of polypharmacy further exacerbates ED complaint in the ageing population.

The proposed pathophysiologic mechanisms responsible for ED in ageing males are likely multifactorial. Firstly, there is presence of atherosclerotic vascular changes within the penile vasculature resulting in decreased blood flow in the penis. Alterations in various neurohumoral pathways such as the α_1_-adrenergic receptor expression, adrenergic sensitivity and nitric oxide synthesis as well as key enzymes contribute to impair penile vasodilation [8]. The presence of endothelial dysfunction as evidenced by a reduction in endothelial function occurs steadily with age in males and highlights the association between cardiovascular disease and ED. Furthermore, cardiovascular risk factors such as hypertension, dyslipidemia, diabetes and obesity exacerbate the underlying inflammatory state in the presence of endothelial dysfunction and reactive oxygen species, thereby increasing the predisposition to plaque formation and atherosclerosis [4]. Histological changes observed in the aged penile tissues showed corporal fibrosis, reduced elasticity and compliance, features consistent with venous occlusive dysfunction resulting in difficulty maintaining penile erection. The loss of penile smooth muscle is further exacerbated in the presence of androgen deficiency, which also resulted in dysregulation of the cavernous nerve function [8].

The current approach to ED management should be patient centred and clinicians should consciously adopt the patients’ perspective and respect the values and expectations of the patients and their partners. The goal of history taking is not only about confirming the diagnosis of ED, but also of identifying the possible underlying and reversible or treatable disorders that contribute to ED. The evaluation of ED should identify the specific erectile problem, patient expectation and reasons for consultation, as well as screen and manage cardiovascular risk factors associated with ED [8]. Patients with an intermediate or high risk of cardiovascular disease should undergo cardiovascular assessment prior to initiating ED treatment. Underlying medical conditions such as depression, diabetes, hypogonadism, metabolic syndrome and iatrogenic causes of ED (e.g., medication) should be ruled out and if present, addressed accordingly. Physical examination should focus on general screening for cardiovascular, neurologic and metabolic health status. A focussed genital examination should be performed to elicit any penile deformity (e.g., penile curvature, plaque and sensation) and determine the testicular size as small testes and regression of secondary sexual characteristics may indicate an underlying hypogonadism. Standard routine blood tests should include fasting glucose and lipids, and hormonal profiles, while specialised tests such as penile colour-duplex ultrasound may be obtained to provide further information.

The current treatment strategies for older men with ED involves setting realistic outcome goals and educating the patient and his partner about the various pathophysiology of ED and the associated risk factors [2,3]. Alternative forms of sexual intimacy that do not involve penetrative sexual intercourse and the satisfaction associated with these activities should also be discussed. Patients should be educated on the benefits of lifestyle changes such as weight loss, exercise and quitting smoking as well as optimization of co-existing medical comorbidities [4,8,9]. Current pharmacotherapy involves replacing non-essential drugs which have an adverse effect on erectile mechanism (e.g., diuretics, beta blockers, and antidepressants) and the use of oral phosphodiesterase type-5 inhibitors (PDE5I). The current recommendation advises starting with a maximal dose of PDE5I (i.e., 100 mg for sildenafil, 20 mg for vardenafil and 20 mg for tadalafil) and repeated dosing may be necessary in some men. Patients should be educated on proper dose intervals and to understand the need for sexual stimulation is necessary for the drug to be effective. In older men with ED and late-onset hypogonadism, the addition of testosterone replacement therapy will enhance the efficacy of PDE5I. While the safety profile of oral PDE5I in the older population is excellent, there are certain heart-related precautions for the use of PDE5I such as in men with unstable angina, poorly controlled hypertension and certain arrhythmias. Vacuum erection devices and intracavernosal injection of vasoactive agents are second-line treatment options that can be effective. They are, however, associated with poor compliance rates because of their side effects such as penile pain, bruising and fibrosis [8,9]. Surgical treatment using a penile prosthesis implant is indicated in men with poor erection unresponsive to medical therapy and/or reported significant adverse effects [10]. The three-piece inflatable penile prosthesis best simulates normal penile flaccidity and erection, like a normal penile erection. Old age should not be a limiting factor for penile prosthesis implantation and studies have shown men aged older than 70 years enjoyed similar satisfaction rate and clinical outcomes, comparable to those of younger men [10].

## 3. Sexual Libido and Desire

Sexual desire is perhaps the most important aspect of male sexual function and it drives all sexual acts. Sexual desire is often closely related to the level of testosterone and given the inverse relationship between testosterone and ageing process, low sexual desire is not uncommon among the older and hypogonadal men [11]. Studies showed an age-dependent decline of testicular function among older men [9].

Hypoactive sexual desire is defined as persistent or recurrent deficient or absent sexual fantasies or desire for sexual activity resulting in significant personal or interpersonal distress [12]. This can be related to organic, psychological and/or endocrine factors, and is a relevant determinant of reduced sexual activity at all ages. In addition to the “physiological” decline in sexual drive with ageing, it is likely that the negative effect of age on desire is more related to overall health problems than to ageing itself. Issues such as medical comorbidities (e.g., cardiovascular conditions and depression), erectile dysfunction, physical limitations, partners and environmental issues have a significant impact on sexual desire.

There are many different testosterone formulations available for men diagnosed with male hypogonadism. The selection of a testosterone replacement therapy should be a joint decision made between an informed patient and his physician, considering issues relating to clinical efficacy, bioavailability, safety and patient’s preference for his testosterone product [13]. Testosterone replacement therapy may be particularly useful to improve not only sexual life, but also the overall quality of life in elderly hypogonadal men, because of its positive effect on muscular strength and cognitive functions [14]. Published literature suggests that hypogonadism may play a role in reduced PDE5I efficacy and that a combination of testosterone replacement therapy and PDE5I potentially enhances the overall efficacy in men who were previously unresponsive to PDE5I treatment [14]. However, due to the adverse effects associated with testosterone replacement therapy, pre-treatment screening for parameters related to potential risks of testosterone replacement therapy is paramount. Testosterone replacement therapy is contraindicated in men with polycythemia, prostatic cancer and in cases of mammary cancer. Importantly the long-term clinical efficacy and safety of testosterone replacement therapy in the ageing male has not been established and caution should be exercised especially in the elderly, frail and hypogonadal men. With regular assessments of efficacy, safety and treatment adherence, testosterone therapy can be safely used to improve the well-being of older men experiencing symptoms of testosterone deficiency.

## 4. Ejaculatory and Orgasmic Function

Ejaculatory and orgasmic dysfunctions are not uncommon and these can range from premature ejaculation to delayed ejaculation and anejaculation, as well as reduced orgasmic pleasure and anorgasmia. Men with ED can present with complaints like premature ejaculation as they often ejaculate quickly to mask the erectile dysfunction. In contrast to premature ejaculation, which is more prevalent in younger men, delayed ejaculation is more common in older men [12]. Delayed ejaculation, retarded ejaculation and inhibited ejaculation are probably the least common, least studied and least understood forms of male sexual dysfunction. These sexual conditions can adversely result in a lack of sexual fulfilment for both the man and his partner, as many men associated ejaculation with the sensation of climax or orgasm. Similarly, a decline in orgasmic function is frequently observed in older men [15] with or without ejaculatory dysfunction.

The treatment strategies in premature ejaculation include self-control techniques, psycho-cognitive behavioural therapy and selective serotonin reuptake inhibitors (SSRI) such as dapoxetine. In contrast, delayed or inhibited ejaculation is probably the least common and understood male sexual dysfunction, and it is important for clinicians to address iatrogenic and pathophysiologic causes such as medications (e.g., antidepressants), hypogonadism and vascular or neurogenic disorders.

However, it remains unclear if this decline in ejaculatory and/or orgasmic functions is because of ageing per se or due to changes in lifestyle, psychological or organic causes. It is likely that changes in sexual desire and erectile function invariably affect ejaculatory and orgasmic functions. The current pharmacotherapy to treat ejaculatory and orgasmic dysfunctions is limited and the role of PDE5I and testosterone therapy remains controversial with very limited published data in the elderly population [9]. Unfortunately, most vasculogenic and neurogenic causes of ejaculatory and orgasmic dysfunction are usually irreversible. Appropriate counselling should be undertaken to establish realistic expectations and the patient should be offered various treatment strategies to improve sexual satisfaction with his partner such as lifestyle changes, alcohol consumption and practice techniques that maximise penile stimulation.

## 5. Conclusions

Ageing invariably affects sexual activity and sexual dysfunctions in aged men and is likely to be multifactorial with the development and/or progression of various medical comorbidities including metabolic, cardiovascular and marital problems. A good physical health, regular sexual partner and active sexual lifestyle earlier in life often predict the maintenance of sexual activity in old age.

While decline in the frequency of sexual activity occurs in advancing age, many men continue to pursue an active sexual life. Common prejudices, beliefs and stereotypes depicting sexual activity as inappropriate, repugnant or taboo in older couple can make it difficult for both health professionals and elderly patients to discuss sexuality and sexual function in a healthy manner. The ageing men and women are often confronted with a vast array of psychological, social and environmental changes. It is important for clinicians to address the various psychological challenges confronting the elderly and how they directly or indirectly cause or exacerbate sexual dysfunction.

At present sexual dysfunction in the ageing male is poorly investigated and understood, and current treatment strategies aim at improving sexual desire and erectile function with limited data on ejaculatory and orgasmic dysfunctions. While the current ED therapies may be effective, they are far from perfect and remain unable to address the growing medical needs of our ageing population. Polypharmacy prescription is common in the elderly and many of these medications can cause sexual dysfunction. Physiological changes related to ageing can lead to pharmacokinetic and pharmacodynamic changes in the elderly with multiple drug interactions and are of particular relevance because they can reduce clinical efficacy (and treatment compliance) and the elderly are more susceptible to drug adverse effects. New hope may derive from the use of newer agents and multimodal therapy to address various biopsychosocial factors. Innovative and novel therapy such as stem cell or regenerative therapy may revolutionise treatment and hope for many older men.

Clinicians should use this opportunity to screen older men for chronic medical conditions such as hypogonadism, depression, cardiovascular and metabolic diseases. Clinicians need to be sensitive and mindful when communicating and addressing sexual problems in older men, with respect to sexual interest and activity among different individuals.

## Appendix A

**Summary box: Practical approach to male sexual dysfunction**

1. Be aware of sexuality and sensitive about sexual problems in the older aged men.
2. Improved communication skills and sensitivity with respect to individual differences in sexual interest and activity.
3. Setting realistic outcome goals and educating the patient and his partner about various pathophysiology mechanisms responsible for male sexual dysfunction and its associated risk factors.
4. Screen older men for chronic medical conditions such as hypogonadism, depression, cardiovascular and metabolic diseases.
5. Education on the benefits of lifestyle changes such as weight loss, exercise and quitting smoking as well as optimization of current medical comorbidities.
6. Treatment options for erectile dysfunction (ED) include management and optimisation of medical comorbidities, oral phosphodiesterase type-5 inhibitors, vacuum erection devices and intracavernosal injection of vasoactive agents. Penile prosthesis implant should be offered in suboptimal erection and/or medically refractory ED.
7. Hypogonadism is associated with decreased sexual desire and the selection of testosterone replacement preparation should be a joint decision made between an informed patient and his physician, taking into account issues relating to bioavailability, safety, tolerability, efficacy and preference of each testosterone product.

**Treatment strategies for various male sexual dysfunctions in the ageing male**

1. Screen for chronic medical conditions such as hypogonadism, depression, cardiovascular and metabolic diseases; exclude iatrogenic or reversible causes; and optimise existing medical comorbidities.
2. Psychosexual counselling (including partner counselling) to set realistic outcome goals.
3. Erectile dysfunction:
  - Phosphodiesterase type-5 (PDE5) inhibitors;
  - Vacuum erection device;
  - Intracavernosal injection therapy;
  - Penile prosthesis implant.
4. Sexual libido and hypoactive sexual desire:
  - Testosterone replacement therapy.
5. Ejaculation and orgasmic disorders:
  - Premature ejaculation—self-control technique, psycho-cognitive behavioural therapy and selective serotonin reuptake inhibitor (SSRI);
  - Delayed ejaculation or anorgasmia—testosterone replacement therapy and pelvic floor muscle therapy.
